# Re: “Valuing the evidence on manual therapy in radius fractures: comments on the study by Horoz and colleagues”

**DOI:** 10.1590/1806-9282.20260545

**Published:** 2026-09-07

**Authors:** Levent Horoz, Basak Cigdem-Karacay, Ismail Ceylan, Halil Alkan

**Affiliations:** 1 Kirsehir Ahi Evran University, Faculty of Medicine, Department of Orthopedics and Traumatology – Kırşehir, Turkey.; 2 Kirsehir Ahi Evran University, Faculty of Medicine, Department of Physical Medicine and Rehabilitation – Kırşehir, Turkey.; 3 Kırsehir Ahi Evran University, School of Physiotherapy and Rehabilitation – Kırşehir, Turkey.; 4 Mus Alparslan University, Faculty of Health Science, Department of Physiotherapy and Rehabilitation – Muş, Turkey.

Dear Editor,

As authors, we read with interest the views of Estrada and Zarria in their letter entitled “Valuing the evidence on manual therapy in radius fractures: comments on the study by Horoz and colleagues” regarding the individualization of Mobilization with Movement (MWM) to optimize its effectiveness in postoperative rehabilitation^1^.

Early mobilization is beneficial for operated distal radius fractures^2^. However, various rehabilitation modalities, compared with early mobilization, have shown limited benefits^3,4,5^. The primary mechanism of action of one such manual therapy method, MWM, is to improve biomechanical function by correcting joint malalignment^6^. A recently published meta-analysis on manual therapy showed that, while studies with both strong and low grade evidence indicated that adding MVM is beneficial for reducing edema and improving functional scores in distal radius fractures, it did not affect range of motion^7^. When examining the studies evaluated in this meta-analysis, including Gutiérrez-Espinoza et al., it is evident that the most common treatment modalities for distal radius fractures are closed reduction and casting or percutaneous pin fixation^7^. Studies have shown that both closed reduction plaster cast immobilization and pin fixation procedures require 4–6 weeks of immobilization after surgery; however, in our study, rehabilitation was initiated in the second week following the removal of the plaster cast. We believe that the success of manual therapy methods stems from their application in cases of long-term immobilization. In our study, we believe the limited superiority in the group that underwent MWM is due to both groups being mobilized early. There is only one study investigating the effectiveness of manual therapy in distal radius fractures treated with volar plates^8^. In this study conducted by Tomruk et al., MWM demonstrated several advantages due to the initiation of MWM in the intervention group after 10 days, and the application of the standard rehabilitation program described in the literature in the control group^9^. In our study, the standard rehabilitation program described in the literature^9^ was tailored to each patient to enable early rehabilitation. We believe the limited benefits in the MWM group were due to the early rehabilitation program applied in the control group. In the study conducted by Tomruk et al.^8^, patient-specific modifications were made to the treatment in the MWM group, rather than applying it as a standard. In our study, however, the treatment in the MWM group was standardized in line with the literature to minimize bias. We believe that another factor contributing to the discrepancy between our results and those reported by Tomruk et al.^8^ is the difference in measurement intervals. Our study results show that MWM has very limited advantages compared to standard early rehabilitation and mobilization. We believe that MWM application will be more effective in distal radius fractures with alignment involvement as a result of long-term immobilization.

## Data Availability

The datasets generated and/or analyzed during the current study are available from the corresponding author upon reasonable request.
